# Neural Mechanisms of Hierarchical Planning in a Virtual Subway Network

**DOI:** 10.1016/j.neuron.2016.03.037

**Published:** 2016-05-18

**Authors:** Jan Balaguer, Hugo Spiers, Demis Hassabis, Christopher Summerfield

**Affiliations:** 1 Department of Experimental Psychology, University of Oxford, Oxford OX1 3UD, UK; 2 Department of Experimental Psychology, University College London, London WC1E 6BT, UK; 3 Google Deepmind, London EC4A 3TW, UK

## Abstract

Planning allows actions to be structured in pursuit of a future goal. However, in natural environments, planning over multiple possible future states incurs prohibitive computational costs. To represent plans efficiently, states can be clustered hierarchically into “contexts”. For example, representing a journey through a subway network as a succession of individual states (stations) is more costly than encoding a sequence of contexts (lines) and context switches (line changes). Here, using functional brain imaging, we asked humans to perform a planning task in a virtual subway network. Behavioral analyses revealed that humans executed a hierarchically organized plan. Brain activity in the dorsomedial prefrontal cortex and premotor cortex scaled with the cost of hierarchical plan representation and unique neural signals in these regions signaled contexts and context switches. These results suggest that humans represent hierarchical plans using a network of caudal prefrontal structures.

**Video Abstract:**

## Highlights

• Humans represent plans in a hierarchical fashion, over contexts as well as states
• Hierarchical plan complexity is encoded in caudal prefrontal cortex
• Ventromedial prefrontal cortex and hippocampus encode proximity to a goal state
• The current context can be decoded from the dorsomedial prefrontal cortex

## Teaser

To represent plans efficiently, states of the world can be clustered hierarchically into “contexts”. Balaguer et al. show that as humans navigate a virtual subway environment, fMRI signals encode the complexity of a hierarchical plan.

## Introduction

By forming and executing plans, humans can engage in complex behaviors such as preparing a cup of coffee or organizing a trip to London. When asked to perform multistep tasks such as these, patients with lesions to the prefrontal cortex (PFC) often exhibit disordered action sequences that fail to achieve the specified goal (Owen et al., 1990, Shallice, 1982, Shallice and Burgess, 1991), and hippocampal patients have difficulty imagining the future states entailed (Schacter et al., 2012). Moreover, functional neuroimaging has confirmed the involvement of human prefrontal and limbic structures in forming and executing plans, particularly in spatial environments (Howard et al., 2014, Schacter and Addis, 2007, Unterrainer and Owen, 2006). Nevertheless, linking these macroscopic neural findings to the underlying computational mechanisms that subserve planning remains an open challenge for psychologists and neuroscientists.

Planning is often described as mental exploration of a network of interlinked, internally represented episodes (or “states”). According to one conception, future states belong to a decision “tree” in which each node is a decision point and each branch a possible response. Plans are representations of trajectories through the tree, selected on the basis of their long-term cumulative outcome (Daw et al., 2005, Daw et al., 2011, Huys et al., 2012, Russell and Norvig, 1995). Computer-based algorithms have successfully exploited this strategy to achieve expert levels of performance in board games such as chess and weiqi (Go) (Silver et al., 2016). However, because the number of possible action sequences grows exponentially with each additional step in the planning horizon, this approach is computationally intractable in many natural environments (Gershman et al., 2015). For example, a visitor would probably not plan a trip to London by envisaging every unique interim step en route to the destination, but might rather imagine attaining only a subset of key states, such as reaching an airport or other transport hub.

**Figure 1:**
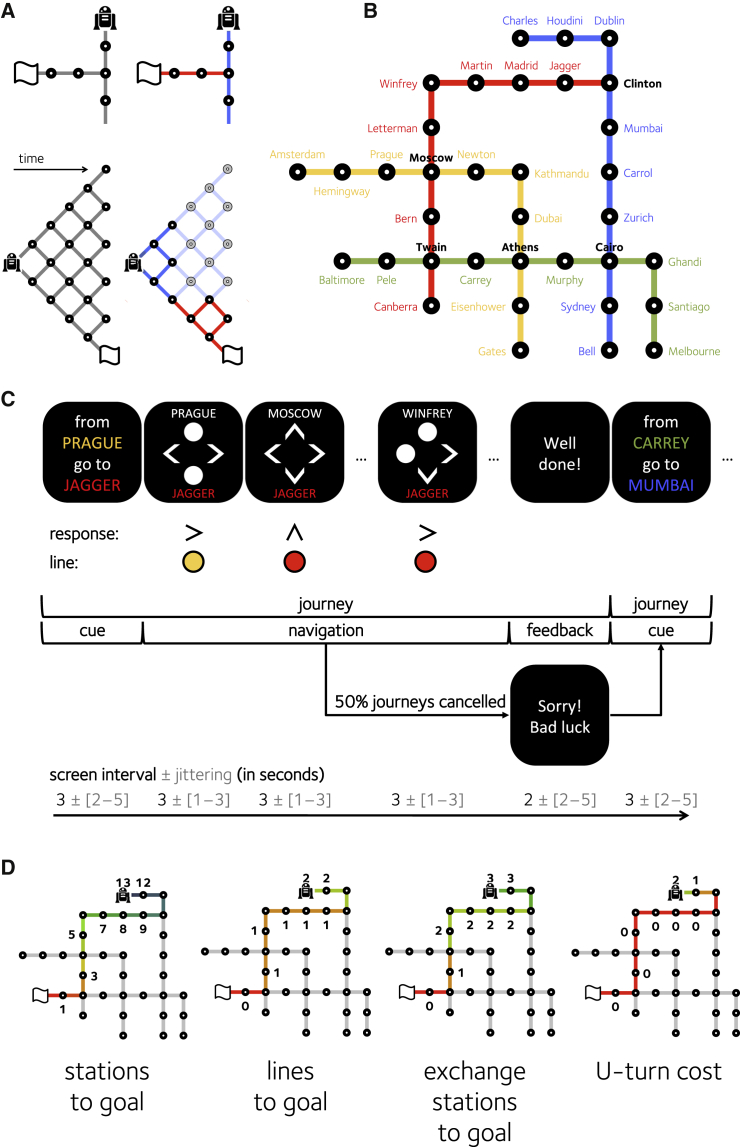
Task and Design (A) Schematic representation of planning under a flat (left) and hierarchical (right) policy. Each node from left (start state, shown by the robot) to right shows a possible state (i.e., station) that could be visited. The flag indicates the destination station. A hierarchical policy allows the agent to “chunk” the maze into contexts (here, a red line and a blue line). This in turn reduces the cost of planning and plan representation. (B) The subway map that participants navigated. The map was rotated and the line colors and station names were shuffled between participants. Participants only saw the map during training. (C) A schematic depiction of the sequence of events (trials) that occurred on an example journey. The names at the top and bottom of the screen refer to the current and destination stations, respectively. The responses (arrows) and lines (colored dots) were not shown to participants. Timings (in seconds) for the various events are shown below. (D) Examples of how the various distances were calculated for an example map: *D*_*S*_ (stations to goal), *D*_*L*_ (lines to goal), *D*_*X*_ (exchange stations to goal), and *D*_*U*_ (U-turn cost). The numbers and blue-red colormap show the distance in each metric that was used to estimate the cost of planning. The robot shows the start point, and the flag shows the destination station.

In machine learning and computational neuroscience, it is widely recognized that the computational demand associated with planning can be reduced by exploiting hierarchical structure in the environment, with states clustered into larger “contexts” (Badre et al., 2010, Botvinick et al., 2009, Koechlin and Jubault, 2006, Sutton and Barto, 1998). To understand how a hierarchical representation may alleviate the computational burden of planning, consider a metropolitan rail (subway) network, in which stations (i.e., states, e.g., King’s Cross and Oxford Circus) are organized into lines (i.e., contexts, e.g., the Victoria Line; see Figure 1A). Unlike planning in a “flat” (non-hierarchical) environment, plans formed in a hierarchical environment need not specify each and every state linking the current position and goal. Rather, it is sufficient to identify the current context and the (termination) conditions that allow the next context to be reached; for example, when planning a journey from Marble Arch to King’s Cross on the London Underground, one should “take the Central Line to Oxford Circus, and from there, switch to the Victoria Line”. Humans seem to represent locations hierarchically in spatial memory: for example, we have a bias to judge cities belonging to a common region (e.g., Nevada) as geographically closer than those crossing a region boundary (Newcombe and Liben, 1982, Stevens and Coupe, 1978). Regionalization may also influence navigational strategy: during wayfinding, humans prefer routes that permit a context boundary to be crossed earlier rather than later (Wiener and Mallot, 2003). In machine learning, states that offer privileged access to a new context (such as Oxford Circus allowing access to the Victoria Line) are considered “bottlenecks,” and hierarchical learning models successfully predict that visiting these should elicit unique patterns of behavior and neural activity (Holroyd and Yeung, 2012, Ribas-Fernandes et al., 2011, Solway et al., 2014).

Here, thus, we taught participants to navigate a novel subway network in which stations (states; e.g., Mandela and Budapest) were organized hierarchically into lines (contexts) defined by their color (Figure 1B). Following training, participants were asked to complete journeys within the network without viewing the map, pressing keys to move from one station to another. We analyzed behavior and fMRI data in order to determine whether humans represented plans in a hierarchical fashion (over lines or contexts) or a flat fashion (over stations or states). On the neural level, an extensive literature has implicated both the medial and lateral PFC in planning on multistep decision tasks such as the Tower of London (Unterrainer and Owen, 2006), but the relative contribution of these different regions remains unclear. Some studies have found that the BOLD signal in dorsolateral PFC scales with the number of moves required to attain goal state (van den Heuvel et al., 2003, Wagner et al., 2006), but neural structures encoding hierarchical plan complexity have yet to be identified. One theoretical perspective has suggested that the dorsomedial PFC (dmPFC) may play a particular role in representing contextual information for future behavior (Holroyd and Yeung, 2012). During passive observation of trajectories through a structured environment, the dmPFC is less active at bottleneck states (Schapiro et al., 2013), but by contrast, a more caudal medial prefrontal region shows a positive “pseudo-reward” signal when a subgoal is attained (Ribas-Fernandes et al., 2011). It thus remains unclear how the medial and lateral PFC might contribute to hierarchical planning.

To preview our findings, we identified two frontal cortical regions that encoded the cost of representing a hierarchical plan: a bilateral anterior premotor region and the dmPFC. These regions also became differentially active at bottleneck states (“exchange” stations, where participants could switch from one context to another). Using multivariate analyses, we found that the dmPFC additionally encoded or monitored the current context (i.e., the subway line that was currently being taken), a key quantity that is required for executing a hierarchical plan. By contrast, the rostromedial PFC and hippocampus encoded the proximity to a goal state. Together, these findings suggest that during planning, humans encode the subway network and formulate plans in a hierarchical fashion.

## Results

### Task Summary

The task is depicted in Figure 1C. Each journey began at a pseudo-randomly chosen station (see Experimental Procedures). On each trial, the names of the destination and current stations were shown, and participants pressed one of four buttons (north, south, east, or west) to move to an adjacent station, which was then shown on the next trial. Their goal was to navigate through the subway map from the start station to the destination station (these successive trials comprising a “journey”). During an initial training session, lines were associated with colors (red, green, yellow, and blue), but at scanning, all color information was removed. Successful journeys were rewarded with financial incentives, but there was a small, but constant, probability that journeys were “cancelled” on each trial and the reward was unavailable, motivating participants to make journeys in the shortest possible number of trials. Participants carried out 88.8 ± 2 journeys in total, each consisting of an average of 5.5 ± 0.06 trials. Of these, 78.3% were performed “optimally” (i.e., when all responses decreased the distance to goal in number of stations). Of the remainder, 15.2% contained at least one action that led participants further away from the goal; these responses were made more slowly (t_19_ = 7.56, p < 0.000001). Additionally, 9.0% of journeys included at least one missing response (when subjects failed to respond on time and remained in the same station as in the previous trial).

### Behavior: The Cost of Plan Representation

The complexity (or description length) of representing a flat (non-hierarchical) plan is proportional to the number of remaining states (here, stations) that must be traversed to reach the goal (here, destination station). By contrast, in a hierarchical plan, this cost scales with the remaining number of contexts that must be traversed for the goal to be attained. We thus began by defining measures of plan complexity that might be computed by participants under flat and hierarchical policies. First, we calculated, on each trial, the number of steps (stations) that remained to be traversed before the goal was reached, assuming a shortest path trajectory (D_S_). This represents plan complexity under a flat policy (see Figure 1D, leftmost). Next, we calculated the number of contexts that remained to be traversed before the goal was reached. Thus, if on the current trial there were only one change of context that would be required to reach the goal, this value would be 1; beyond that context switch, the value would be 0. This quantity D_L_ indexes the cost of a hierarchical policy (Figure 1D, center left). Then, as a control, we computed the distance to goal in number of exchange stations to be traversed. By design, on many journeys, the shortest path involved passing through an exchange station without switching context (Figure 1D, center right). This measure, which we call D_X_, was thus decorrelated from D_L_ (for details of the correlation among distance measures, see Table S3). Finally, we computed another cost, which represented the number of steps that had to be taken away from the goal (in cityblock space) in order to reach it by the shortest path. Thus, this measure, which we call the U-turn cost (or D_U_), was high for paths that required “doubling back” (Figure 1D, rightmost).

**Figure 2:**
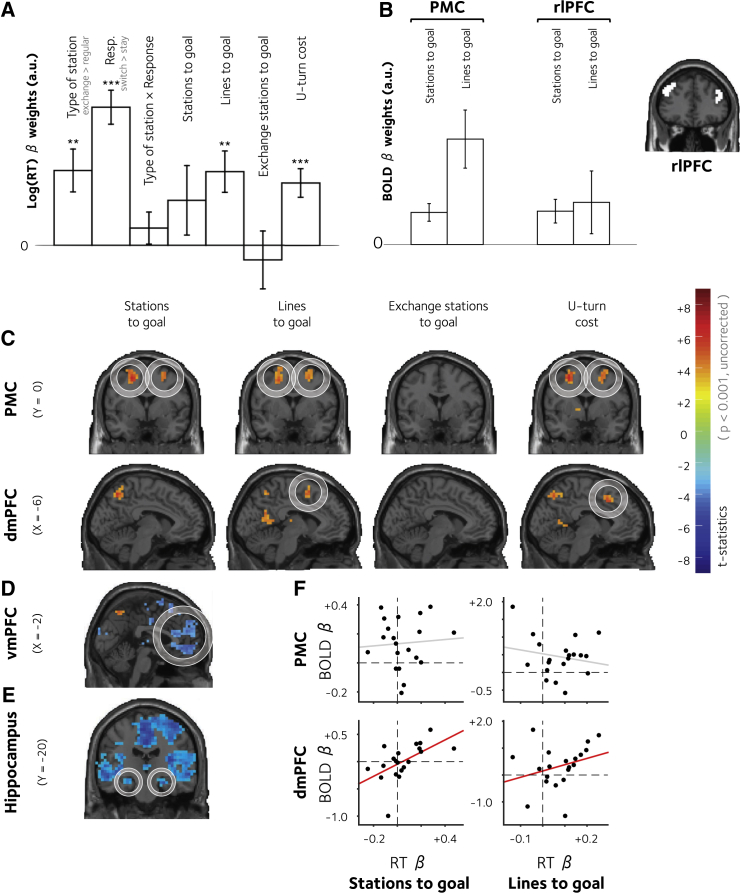
Behavioral and Neural Costs of Flat and Hierarchical Planning (A) Regression coefficients (mean ± SEM across participants) showing the slope of the predictive relationship between experimental variables (including distance estimates) and log RTs. (B) Parametric responses (mean ± SEM) to *D*_*S*_ and *D*_*L*_ in the PMC and rlPFC. There is a significant condition × region interaction. The rlPFC ROI is shown on the right. (C) Encoding of the four plan complexity measures (GLM1) in the lateral (coronal view; upper) and medial (sagittal view; lower) frontal cortices, rendered onto a template brain, thresholded at p < 0.001 uncorrected. (D) Correlation with proximity to goal (GLM1) in the vmPFC. (E) Correlation with proximity to goal (GLM2) in the hippocampus. The activations are shown that exceed p < 0.001, uncorrected. (F) Correlation between parameter estimates linking log(RT) to plan complexity in units of station (left) and lines (right), with beta values encoding the corresponding distance measure in the PMC (upper) and dmPFC (lower). The dots correspond to individual subjects. The lines are to best linear fits for significant (red) and non-significant (gray) correlations, respectively. The significant regions within a circle survived multiple comparisons correction.

We then used linear regression to ask whether (log) response times (RTs) during navigation were sensitive to the complexity of the plan as indexed by *D*_*S*_, *D*_*L*_, *D*_*X*_, and *D*_*U*_. Critically, this analysis yielded significant positive coefficients for number of lines to goal (D_L_: t_19_ = 3.46, p = 0.003) and for the U-turn cost (D_U_: t_19_ = 4.26, p < 0.001; see Figure 2A). When these predictors competed for variance within a single regression, however, the number of stations to goal failed to predict RTs (D_S_: t_19_ = 1.26, p = 0.223), as did the number of exchange stations (D_X_: t_19_ = −0.49, p = 0.628). This finding suggests that the main costs of representing the plan were contextual or structural aspects of the subway map, rather than the number of unique steps required to reach the destination station. This supports the view that plans are formed and executed in a hierarchical fashion.

We defined stations as “regular” (i.e., within a single line; e.g., Madrid in Figure 1B) and exchange (i.e., bottlenecks, occurring at the intersection between lines, e.g., Clinton). Moreover, responses were classified as either stay (i.e., travel in the same direction as the previous step) or switch (i.e., change the direction of travel). These factors were orthogonal in our paradigm, because regular stations sometimes required a direction switch, as when a single line turned a corner (e.g., Kathmandu in Figure 1B), but participants could also pass through exchange stations without switching response (e.g., when passing through Moscow en route from Winfrey to Bern). This feature of our design thus allowed us to further include, in the above regression, separate binary predictors encoding station type (exchange versus regular) and response type (switch versus stay). We observed a main effect of station type (exchange > regular; t_19_ = 3.40, p = 0.003) and of direction (switch > stay; t_19_ = 7.92, p < 0.001). The interaction between station type and response type was not significant (t_19_ = 1.05, p = 0.309). Mean RTs in each condition are plotted in Figure S1.

### Neural Cost of Plan Representation

Next, we sought to identify in the brain imaging data the neural costs of representing flat or hierarchical plans. In this analysis and all that follow, all reported results survive correction for multiple comparisons using a false discovery rate (FDR) with an alpha of p < 0.05, unless otherwise noted. We built a design matrix (GLM1) with regressors encoding the various indices of distance to goal introduced above (*D*_*S*_, *D*_*L*_, *D*_*X*_, and *D*_*U*_; Figure 2C). Examples of how these distances were computed are shown in Figure 1D. Regressing this design matrix against BOLD data, we found that a dmPFC (BA8/32) responded positively to the cost of plan representation in units of both lines (peak: −6, 8, 58; t_19_ = 5.21, p < 0.0001) and the U-turn cost (peak: −2, 12, 46; t_19_ = 5.63, p < 0.00001). Critically, in GLM1 (when all four regressors competed to explain variance in BOLD activity) no dmPFC voxels were sensitive to the distance to goal in terms of number of stations.

In the lateral PFC, we observed a similar pattern of BOLD signals in an anterior premotor region (premotor cortex) that straddled BA6 and BA8, where BOLD activity scaled with *D*_*L*_ (left peak: −26, −8, 54; t_19_ = 6.58, p < 0.000001 and right peak: 30, 4, 66; t_19_ = 4.99, p < 0.0001) and *D*_*U*_ (left peak: −26, 4, 54; t_19_ = 6.51, p < 0.000001 and right peak: 26, 8, 46; t_19_ = 6.30, p < 0.000001). Here, we also observed an effect of distance in number of stations, *D*_*S*_ (left peak: −22, −8, 50; t_19_ = 6.62, p < 0.000001 and right peak: 30, 4, 58; t_19_ = 6.39, p < 0.000001). Notably, the number of exchange stations between the current position and the goal (*D*_*X*_) failed to show any consistent effect at the group level. In other words, these regions encoded the cost of representing a plan in units that reflected the structure of the subway map, over and above any encoding of the distance to goal.

Previous neuroimaging studies have noted that BOLD signals in the rostrolateral PFC (rlPFC) scale with the number of moves that are required to solve the Tower of London task (van den Heuvel et al., 2003, Wagner et al., 2006), equivalent to our *D*_*S*_ measure. To permit direct comparison with past studies, we created a new GLM (GLM2) that included only *D*_*S*_ (alongside other nuisance quantities; see Experimental Procedures), omitting the distance regressors in units of lines, exchange stations, or the U-turn cost. Consistent with previous work, this analysis identified not only the premotor cortex (PMC), but also a portion of bilateral rlPFC (left: −42, 32, 34; t_19_ = 7.87, p < 0.000001 and right: 42, 40, 34; t_19_ = 4.81, p < 0.0001; see Figure 2C). Plotting the average beta parameters across the cohort for *D*_*S*_ and *D*_*L*_ confirmed that the PMC, but not the rlPFC, encoded the cost of a hierarchical plan, as demonstrated by a region (PMC and rlPFC) × distance (*D*_*S*_ and *D*_*L*_) interaction (F_1,19_ = 4.71, p < 0.05; see Figure 2B).

### Proximity to Goal

Consistent with previous findings (Howard et al., 2014), using GLM1, we also observed a signal that reflected a negative correlation with distance in stations to goal (D_S_) in the ventromedial PFC (vmPFC, peak: 10, 48, −6; t_19_ = 5.80, p < 0.00001; in other words, this region became more active the closer to the goal). In this region, distance was encoded in units of stations only, with no evidence for encoding of hierarchical distance (Figure 2D). Including only *D*_*S*_ (GLM2) identified a number of other regions, including the hippocampus, where BOLD signals have previously been found to scale with distance to goal during navigation (Howard et al., 2014). In our task, the hippocampus reflected distance to goal bilaterally in the same direction as the vmPFC (Figure 2E). A full range of regions that correlated with each of these distance estimates is reported in Tables S1 and S2.

### Correlation of Neural and Behavioral Costs across the Cohort

Next, we aimed to understand the relationship between the neural and behavioral effects so far observed (see Figure 2F). For each measure of planning cost (*D*_*S*_, *D*_*L*_, *D*_*X*_, and *D*_*U*_), we calculated the correlation across the cohort of participants between its influence on RT (regression coefficient from Figure 2A) and its influence on BOLD signals in (1) the PMC and (2) the dmPFC. We found the correlation was significant in dmPFC for both distance in number of stations (D_S_: R = 0.6, p < 0.005) and in number of line changes (D_L_: R = 0.39, p < 0.05). However, neither of these correlations was significant in the PMC (D_S_: R = −0.05, p = 0.57 and D_L_: R = 0.07, p = 0.379). No brain-behavior correlations were observed in either region for *D*_*X*_ or *D*_*U*_. However, we did observe a correlation between the behavioral cost of *D*_*U*_ and the encoding of *D*_*U*_ in a dlPFC region shown in Figure S4 (D_U_: R = 0.33, p < 0.05 one-tailed).

### Neural Signals Associated with Bottleneck States

**Figure 3:**
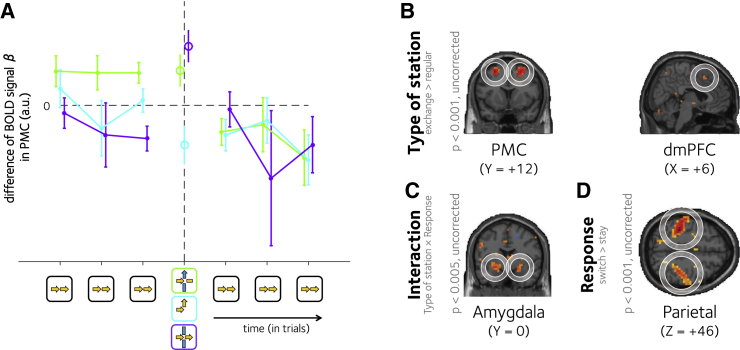
BOLD Responses to Bottleneck States (A) BOLD signal β values (mean ± SEM) from single-trial GLM approach in the PMC on three regular stations preceding (leftmost points) and following (rightmost points) a context switch (green lines), an exchange station without line change (purple lines), or an elbow station (cyan lines). The activation at the context switch, exchange station, or elbow are shown with a single point in the corresponding color. The averaged BOLD signal β in regular stations is represented by the horizontal dashed line. (B) Voxels responding to the main effect of station type (exchange > regular) in the PMC (left) and dmPFC (right). (C) Voxels in the amygdala responding to the interaction between station type and response. (D) Voxels in the parietal cortex responding to the main effect of response switch. The coordinates in MNI space are provided under each slice. The significant regions within a circle survived multiple comparisons correction.

The analyses described above suggest that both dmPFC and PMC encoded the hierarchical cost of representing a plan, over and above any cost of plan representation computed in units of discrete states. Next, we investigated neural signals in these regions more closely, by plotting the activity that accompanied the moment in which a bottleneck state occurred, when participants were offered the opportunity to switch from one context to another. We once again capitalized on the factorial design of our task, asking if there were unique neural signals that varied with station type (exchange > regular, now including all trials; Figure 3B). This analysis also included a regressor encoding *D*_*S*_, as well as a further nuisance predictor that signaled whether the action chosen was optimal or not (GLM2).

We observed increases in BOLD signals associated with exchange stations in both the dmPFC (peak: 6, 16, 46; t_19_ = 4.09, p < 0.001) and PMC, overlapping with the region described above (left peak: −26, 8, 54; t_19_ = 7.24, p < 0.000001 and right peak: 26, 12, 54; t_19_ = 6.56, p < 0.00001). Across the subject cohort, the strength of this latter neural effect predicted the RT difference between exchange and regular stations (r = 0.40, p < 0.04), but not between switch and stay trials (p = 0.70). A further effect of exchange > regular stations was observed in a more anterior prefrontal region, in bilateral BA 46 (left peak: −42, 24, 30; t_19_ = 4.48, p < 0.0001 and right peak: 46, 32, 22; t_19_ = 5.38, p < 0.0001).

Next, we plotted how the BOLD signal varied on those regular stations that both preceded and followed an exchange or an elbow station. A brain region encoding the hierarchical representation of a plan might be expected to show tonically higher BOLD signals in the trials preceding an exchange station (where the cost of plan representation in units of lines remains high), followed by a reduction immediately after context switch (where the computational burden is reduced). In Figure 3A, we plot the BOLD signal in the PMC region (extracted from the main effect of type of station) on regular stations that precede and succeed a context switch (green lines). An elevated BOLD signal is visible on those trials preceding a context switch, after which it drops off sharply (comparison between preceding and succeeding: t_19_ = 3.24, p < 0.003). Of note, a similar drop is not observed when the same analysis is conducted on stations that precede or succeed an exchange station without a context switch (purple lines; p > 0.9) and only a modest drop follows an elbow station (t_19_ = 1.87, p < 0.05, one tailed). These effects were qualified by the interaction of type of station and type of response on the difference of signal (preceding and following) around each condition: F_1,19_ = 5.44, p < 0.04. In other words, the average BOLD signal in PMC observed was higher on trials before than after a context switch, consistent with a hierarchical representation of the plan. We additionally found a main effect of type of response: F_1,19_ = 4.61, p < 0.05, indicating that participants also anticipated making a response switch. Signals from the dmPFC followed a similar pattern, although the interaction failed to reach significance. An equivalent analysis for RTs is shown in the Supplemental Information (Figure S3).

### Neural Signals Accompanying Response Switch and Context Switch

Behavioral data indicated that there was a unique cost incurred when participants switched context, i.e., at exchange stations requiring a response switch. In the fMRI data, we observed a comparable interaction between type of station and response switch in a cluster of voxels straddling the amygdala and putamen (left peak: −26, 0, −10; t_19_ = 4.46, p < 0.001 and right peak: 22, 4, −14; t_19_ = 5.20, p < 0.0001), as well as an extrastriate region on the lingual gyrus (peak: 26, −68, −6; t_19_ = 5.16, p < 0.0001), corresponding to area V4 where responses to color are often observed (Zeki and Marini, 1998). Plotting parameter estimates for these regions showed that this interaction was driven by higher BOLD signals for those trials where participants switched from one context to another (Figure 3C). However, we interpret these results with caution, because they failed to reach the threshold required for correction using an FDR threshold. Finally, we also observed strong activations in the parietal cortex that predicted whether participants switched direction or not (left peak: −38, −32, 46; t_19_ = 10.8, p < 0.000000001 and right peak: 54, −24, 34; t_19_ = 8.39, p < 0.0000001; Figure 3D).

### Encoding of Current Context

**Figure 4:**
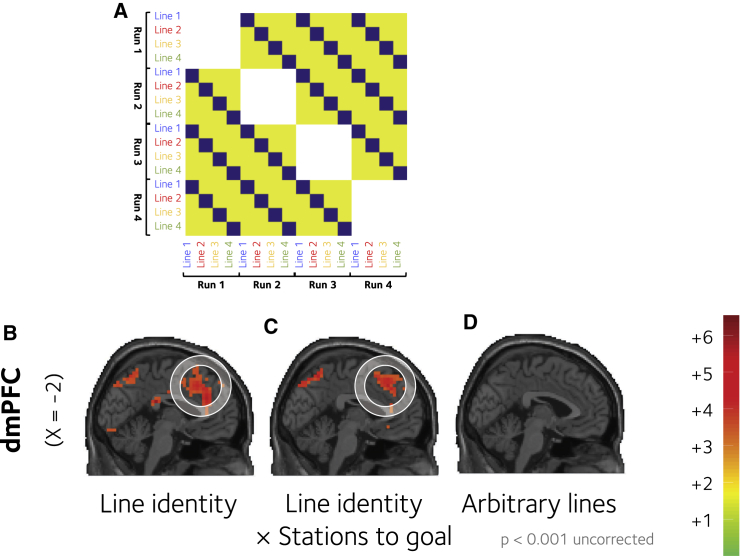
Encoding of Context in Multivariate BOLD Signals (A) A depiction of the predicted representational dissimilarity matrix that was used to identify brain regions where the similarity structure was greater within than between contexts. The blue (and yellow) squares represent low (high) dissimilarity, respectively for independent pairs of scanner runs and lines (x and y axis). (B) The results of the RSA identifying voxels encoding context, i.e., where multivoxel pattern dissimilarity was greater between than within contexts (lines), identified using a searchlight approach. (C) Voxels where the pattern encoding the parametric distance to goal (in units of station) was more different between than within contexts (lines). (D) The results of the control analysis for (B) involving shuffled stations-line assignments. An additional control analysis was performed to assert that the effect was not driven by line orientation (see Figure S2). The significant regions within a circle survived multiple comparisons correction.

To execute a hierarchical plan, an agent must be able to identify and represent the current context, in addition to the current state (i.e., on the London Underground, to know that one is on the Victoria Line, not just that one is at Green Park station). We thus used a multivariate analysis technique known as representational similarity analysis (RSA) to identify brain regions in which the patterns of BOLD signal over voxels was more similar across runs within a single subway line than between two different lines (using unsmoothed data; see Experimental Procedures for details; Figure 4A). In the scanner, no indication was given as to the subway line currently being visited, and so any significant voxels must reflect an abstract encoding of the context from memory. In conjunction with a whole-brain “searchlight” approach, this analysis once again identified the dmPFC as a region where the current context was represented (peak −10, 8, 54; t_19_ = 7.49, p < 0.000001; Figure 4B). No evidence for context encoding in the PMC was found, although evidence was found in other regions, including more anterior portions of the PFC in BA9 (left peak: −30, 44, 34; t_19_ = 5.32, p < 0.0001 and right peak: 34, 44, 30; t_19_ = 4.9, p < 0.001).

The analyses above indicated that the dmPFC encodes distance to goal in units of lines and U-turns. It could be, thus, that the pattern encoding of this quantity may depend on the current line, providing evidence for a distinct computational cost within each context. We thus repeated our RSA, but using not the raw BOLD signal observed at each station, but the parametric encoding of distance to goal (in stations). The pattern of encoding of distance to goal was also more similar within lines than it was between lines in the dmPFC (2, 20, 54; t_19_ = 5.38, p < 0.0001); it is shown in Figure 4C.

RSA can yield spurious results when trials assigned to each category are not fully temporally decorrelated, and so we conducted this analysis between runs (e.g., measured the similarity between line a on run1 and line b on run 2). We additionally conducted a control analysis in which the assignments between stations and lines were shuffled; this yielded no significant results (Figure 4D).

Finally, subway lines contained long straight sections, and so we were concerned that RSA of context might have captured similarity associated with travel in a common direction, unrelated to context per se. To test this, we conducted another RSA using the same approach, but searched for regions where multivoxel patterns were more similar within than between directions (north, south, east, and west). No activations were observed in the medial PFC, but a large cluster of significant voxels was found in the left motor cortex (Figure S2).

## Discussion

The behavior of humans and other animals is controlled at least in part by a “model-based” control system that learns the structure of the world and organizes sequential behavior in pursuit of future goals (Daw et al., 2005, Dickinson and Balleine, 2002, Dolan and Dayan, 2013, Schoenbaum et al., 2009, Tolman, 1948). Recent work has begun to address the neural and computational substrates underlying the model-based decision-making by constructing “two-step” decision tasks in which cached state-action values and explicit forward search strategies make opposing predictions about behavior and brain activity (Daw et al., 2011, Gläscher et al., 2010, Wunderlich et al., 2012). However, these studies sidestep one key theoretical challenge associated with model-based approaches, namely, how to organize behavior over multiple future states without incurring a prohibitive computational cost. Human cognition has evolved to meet this challenge, as exemplified by our ability to form and follow plans over multiple timescales, for example when finding an efficient route to run a series of errands, or envisaging a future career path and taking steps toward its fulfillment. Although we have known for decades that planning involves the PFC, to date, very little has been revealed about the computational mechanisms that unfold in these regions during plan formation and execution.

Here, we drew upon a framework that has its roots in cognitive psychology (Miller et al., 1960, Norman and Shallice, 1986), but has most recently inspired advances in machine intelligence (Botvinick et al., 2009, Ponsen et al., 2010). This framework proposes that the space of possible states can be organized and represented hierarchically as a series of clusters or contexts, reducing plan complexity (description length), and affording substantive increases in computational efficiency both at the time of plan formation and plan execution. In the current study, we tested a prediction arising from this hypothesis: that when planning in a complex environment, the cost of representing a plan will be expressed in units of context (or context switch) over and above any cost that is incurred in units of states themselves. Our key finding is that both RTs and neural activity in the caudal frontal cortex encode the cost of representing a hierarchical plan, indicating that they participate in the hierarchical organization of future behavior.

The neural costs observed were identified in two frontal regions: a dmPFC region, falling in the presupplementary motor cortex, that is often found to be sensitive to the difficulty (or conflict) incurred when making a choice (Botvinick et al., 1999), and a lateral frontal that straddles the border between the premotor and prefrontal cortices, in BA6/BA8. Both regions were also active when participants were faced with the opportunity to switch context, at an exchange station or bottleneck, consistent with the finding that the dmPFC responds to subgoal attainment (Ribas-Fernandes et al., 2011). However, across the participant cohort, we observed reliable brain-behavior correlations in only the dmPFC, but not the PMC. In the dmPFC, the strength with which BOLD signals encoded distance to goal in units both of stations and contexts for a given subject predicted his or her corresponding RT cost for those plan complexity measures. We also found that the multivariate pattern of information in the dmPFC (but not PMC) was sufficient to distinguish among contexts, even though the line that was currently visited was never explicitly displayed to participants during the scanning phase. Moreover, we were also able to distinguish context-specific representations of distance to goal in the dmPFC, as if the region encoded separate costs of planning for each individual context. One interpretation of this finding is that the dmPFC is responsible for the translating of a plan into behavior, whereas the PMC participates in maintaining the active plan over the journey. However, we note that those participants showing the strongest flat cost in behavior also showed stronger encoding of this cost in dmPFC neural signals. It may be, thus, that there are some individual differences in the way that dmPFC contributes to computing the cost of planning.

More generally, our findings are consistent with the view that the dmPFC encodes a motivational signal that is extended over time (Summerfield and Koechlin, 2009) and the complementary perspective that the dmPFC encodes “option” values under the framework of hierarchical reinforcement learning (Holroyd and Yeung, 2012). As part of a general role in monitoring the expected value of controlled behavior (Shenhav et al., 2013), the dmPFC may thus encode both the identity and value of a contextual variable over which a particular policy applies, for example, when foraging from different patches (Hayden et al., 2011, Kolling et al., 2012).

The lateral region overlaps with the superior aspect of the caudal dorsolateral PFC identified by Koechlin et al. (2003) as active when actions are selected on the basis of contextual information. The same region is labeled “pre-PMd” by Badre et al. (2010), who found that this region is active when action selection is contingent on a hierarchy of contingencies, rather than a flat series of sensorimotor associations. In this region (as in behavior and the dmPFC signal), the BOLD signal scaled with distance to the destination station in units of context (i.e., lines), but not the metric provided by individual states (i.e., stations). Notably, no such effect was observed in more rostral regions that have previously been implicated in representing plan complexity in multistep problems such as the Tower of London task (van den Heuvel et al., 2003, Wagner et al., 2006). At first glance this finding is surprising, one might have expected more anterior regions to be responsible for representing the higher hierarchical aspects of a complex plan. However, one explanation for this finding is that during hierarchical planning, potentially complex action sequences are “compressed” to a small number of steps (e.g., contexts and context switches) that can then be represented in subsidiary prefrontal regions located more caudally (Koechlin and Summerfield, 2007).

Interestingly, the cost of representing a plan was incurred in units of context, but not in units of response switch. This explains the previous finding that humans seek to reach a new context earlier rather than later during navigation, as doing so reduces the computational burden of plan representations (Wiener and Mallot, 2003). This result additionally suggests that the hierarchical representation of the plan is encoded in terms of its abstract structure, rather than as a succession of macro-actions (e.g., “go straight, then go left”). Nor was the plan encoded in terms of the number of choice points, suggesting that the state space is not chunked purely on the basis of its physical properties (e.g., in terms of segments between choice points), but in a fashion that reflected the more abstract structure that they were encouraged to learn during training. What remains unclear, however, is whether context is represented as a cluster of interlinked perceptual states (i.e., stations on the yellow line), or as a series of macro-policies that dictate pursuit of a goal (e.g., keep going straight on until you reach a given switch point). A hint that participants relied on perceptual representation of context was provided by the finding that voxels in area V4 became active at context switches, as if participants were recalling the color of the new subway line (which was not shown to them during scanning). However, the precise nature of the information that characterizes a context remains an open question. For example, participants might have used information about the spatial organization of the map (the blue line runs from north to south or the red line is north of the green line).

Moreover, both behavior and the PMC also encoded an additional “U-turn” cost, that indexed the extent to which plans involved doubling back toward the current location along a different line. In the planning literature, it has been noted that goal-subgoal conflict—for example, the need to temporarily remove one disc from a peg and subsequently replace it in the Tower of London task—incurs a unique RT cost (Ward and Allport, 1997) and poses a particular problem for patients with lateral prefrontal lesions (Morris et al., 1997). Consistent with this finding, U-turn costs were visible not only in the PMC, but also in lateral prefrontal regions. The existence of a unique U-turn cost in our navigation task demonstrates that participants not only encoded plans in the subway network as a hierarchical series of contexts, but also in terms of the geometry of the map that they saw in the training session.

Although the costs of representing a flat plan were minimal once variance associated with a hierarchical plan had been partialled out, there was one brain region where strong (positive) covariation with number of stations to goal was observed, the vmPFC. Previous theories have speculated that the vmPFC may be among a set of regions that tracks distance to a goal state (Holroyd and Yeung, 2012) and, indeed, the vmPFC is implicated in episodic future thinking (Schacter and Addis, 2007), and has been found to track growing expected reward in decision tasks involving sequential, interdependent choices (Tsetsos et al., 2014). The hippocampus has also previously been found to covary with proximity to goal, but only in virtual reality environments that mimic much more closely the naturalistic experience of navigation (Howard et al., 2014, Viard et al., 2011). Here, we show that the distance to goal representation is present even when current and goal state information is devoid of the rich episodic cues that we normally use to navigate. Critically, however, the hippocampus and vmPFC showed no evidence of a hierarchical signal.

Our analyses focused on the cost of “representing” a hierarchical (or flat plan) as participants navigated through the network. This is a general index of the cost involved in maintaining and monitoring the plan, rather than of recursively searching through all possible nodes of the decision tree (for example, via a breadth- or depth-first algorithm) or “pruning” of unpromising routes to a goal (Huys et al., 2012, Huys et al., 2015). While plan formation may have occurred mainly on presentation of the cue screen stating the start and goal stations, plans may also have been constantly updated and reformed during execution (“replanning”). Indeed, as distance to goal grows, the processing cost of these search operations will grow correspondingly. However, it is not clear that this cost would grow linearly with the number of states or contexts that must be traversed to reach a goal. One limitation of the approach taken here is that we do not have an obvious means to assess how plans are formed prior to or during navigation or to distinguish the neural mechanisms that accompany plan maintenance and monitoring from any replanning that may be occurring. We did examine BOLD signals evoked in response to the cue screen, but they did not show convincing correlations with the various distance metrics or predict the journeys that participants would follow. However, it is unclear whether this null finding is due to a lack of statistical power, owing to the limited number of such trials. Examining the costs incurred at the time of plan formation would be an interesting avenue of research for future studies.

## Experimental Procedures

### Subjects

A total of 22 healthy participants (10 female and 12 male; age 19–34, mean 25.6 years; one was the first author of the study) were recruited into the study in accordance with local ethical guidelines. No participants reported a history of psychiatric or neurological illness, and all had normal or corrected-to-normal vision. Participants were paid £35 for participation in both a practice and a scanner session on two separate days. A monetary incentive of up to £10, proportional to performance, was added to the previous amount. There were two participants that were excluded due to poor performance on the task (more than 20% of the journeys included a move in the wrong direction during the main experiment).

### Stimuli and Task Design

The same subway map was used for all participants, but the names of the stations and the colors of the lines were randomly shuffled, and the map was randomly rotated by 0°, 90°, 180°, or 270° (example shown in Figure 1B). Following training (see below), participants performed the main task, which involved navigating in a virtual subway environment, in the MRI scanner. Each journey involved a start station and a destination station that were randomly selected with the constraint that the journey would require at least one change of line (17.8% of journeys) or one change of direction without changing lines (10.7%) or both (71.5%). Participants navigated through the subway map by pressing buttons (see below). On each trial, there was a constant probability that the journey was cancelled, engineered such that cancellation probability was independent of the length of the optimal journey and led to approximately 50% of journeys being cancelled; cancellation probability was independent of the hierarchical aspects of the task. Overall, 52.9% of journeys were successfully completed. Each journey was rewarded with a monetary value (either one or five virtual coins, signaled during navigation), which were converted to real incentives (normalized to a maximum of £10) that were paid out as a bonus at the end of the experiment. Behavioral performance did not differ as a function of the incentives offered, so we collapsed over this factor for all analyses.

### Procedure

The main task is depicted in Figure 1C. Each journey began with the presentation of a cue screen for 3 s that indicated the starting point and the destination (stations and lines). After a period of 2–5 s (jittered) of blank screen, on each of the successive trials a navigation screen was displayed for 3 s. This screen provided multiple pieces of information: the names of the current and destination stations; the line color of the destination station; the reward at stake for the current journey; the cumulative reward so far; and the cardinal directions (north, south, east, and west) available from the current station. Critically, no information about the current line or about the line associated with each action was shown. At each step, participants had to choose the direction they wanted to take by pressing one out of four buttons. If no key was pressed, the same station was shown again in the next step. Each navigation screen was followed by a blank screen of 1–3 s (jittered); no feedback was provided during navigation.

The journey ended either when the participant reached the destination or when the journey was cancelled. After the journey was finished or cancelled, a feedback screen informed whether the destination had been reached or not and the reward that had been obtained. This screen was displayed for 2 s and was followed by a blank screen of 2–5 s (jittered) before the next cue screen occurred. Participants completed as many journeys as possible in four successive runs buttressed by lead-in and lead-out durations of 10 s and 5 s, respectively. The total scanning time, including anatomical and localizer scans, was around 75 min per participant.

### Training Task

All participants were trained in a separate behavioral session that took place outside the scanner exactly 2 days before the main experimental task. This training session was similar to the main task, with the following exceptions. First, the map (e.g., Figure 1B) was shown for 10 s prior to the start of each journey. Second, participants were allowed unlimited time to respond, moving on to the next screen only after a key press had been initiated. Third, the available actions were shown in the color of the corresponding line, and a picture matching the name of each station was displayed consistently in the background to facilitate the learning of the map. An additional key press (space bar) was required to switch between lines and, during a line switch, an animated clock was shown on screen and a delay of 1 s was imposed. On each journey, the starting and destination stations were selected uniformly, permitting a larger number of possible journeys and, at the end of each journey, a feedback screen informed the participant of (1) the total length of the journey and (2) the minimum length that could have been be achieved (i.e whether their journey had been optimal or not). During training, journeys were never cancelled and no monetary outcomes were associated with successful journeys. Lastly, we introduced ten “quizzes” at homogeneous times during the training session (always between journeys), each including ten “questions” where the current station and the goal were cued, but participants were only required to respond to the first step toward the goal. Participants were informed of the scores obtained at the end of each quiz and were instructed to learn during the whole session as to maximize their scores during the quiz. They completed as many journeys as possible over a period of 45 min.

On the day of scanning, before entering the MRI, participants performed a practice block identical to one of the main task scanner runs. They were allowed to see the map one last time before the beginning of this second training session. Data from this session were not included in the analyses.

### fMRI Acquisition

Magnetic resonance images were acquired with a 3T Siemens VERIO scanner with a 32-channel head coil using a standard echo-planar imaging sequence. Whole-head *T*_*2*_*^∗^*-weighted echo-planar images were continuously acquired with a repetition time of 2 s, echo time of 30 ms. We acquired fMRI data in four runs (∼17 min each) of between 456 and 510 volumes, plus three dummy scans discarded before the analyses. For technical reasons, three participants completed only three runs. Each volume included 64 × 64 × 36 voxels of 3 × 3 × 3 mm. A high-resolution T1-weighted structural image was also obtained (voxel size = 1 × 1 × 1 mm). For standard preprocessing and univariate statistical analyses, we used SPM12 (Wellcome Department of Cognitive Neurology, London, United Kingdom). All other analyses were carried out with custom scripts for Matlab (Mathworks). We also used *XjView* (http://www.alivelearn.net/xjview) to visualize the data and to construct mask images and impose an FDR correction for multiple comparisons (Genovese et al., 2002). For each participant, we first realigned all functional images, then we co-registered (rigid body transformation) the anatomical scan to the mean functional image. We then segmented each subject’s co-registered anatomical scan, using segmented probabilistic maps for gray matter, white matter, cerebro-spinal fluid, bone, soft tissue, and air/background in the Montreal Neurological Institute (MNI) space. The parameters obtained were applied to normalize the subject’s functional scans to the template brain MNI space. Functional images were resampled (3 × 3 × 3 mm voxels) and spatially smoothed (6-mm full-width half-maximum [FWHM] Gaussian kernel). For all analyses, a 128 s temporal high-pass filter was applied to remove low-frequency scanner artifacts. Temporal autocorrelation in the time series data was estimated using restricted maximum-likelihood estimates of variance components using a first-order autoregressive model (AR-1), and the resulting non-sphericity was used to form maximum-likelihood estimates of the activations, consistent with standard approaches in SPM (Penny et al., 2006).

### Behavioral Analyses

We analyzed log reaction times with linear regression as described in the main text and the significant contribution of each regressor was validated through a t test using an alpha of p < 0.05. All regressors and interactions were *Z* scored before being introduced in the regression. The optimal path was obtained through a generalized version of the Dijkstra algorithm that minimized multiple distances, by priority: in number of stations, number of response switches, and number of exchange stations. The U-turn cost was defined as the signed difference between the distance in number of stations and the Manhattan (cityblock) distance: D_U_(a,b) = D_S_(a,b) − |x_a_ − x_b_| − |y_a_ − y_b_|, where (x_i_,y_i_) are the geometrical coordinates of a station *i*, |·| is the absolute value operator, and D_S_ is the distance in number of stations. An illustration of how the various indices of distance to goal were computed is shown in Figure 1D. The original Dijkstra algorithm was based on in-house code.

### Univariate Analyses of Functional Data

All univariate analyses were based on a generalized linear model (GLM) approach. Our GLM included regressors coding for onsets and durations of stimuli or events, which were then convolved with the canonical haemodynamic response function (HRF) and regressed against the observed fMRI data. Scanner runs were concatenated for univariate analyses, and constant terms for each run were included manually. Additionally, motion parameters and the average signal outside of the brain were included as nuisance variables for all GLMs. Group-level statistics were estimated from the individual β patterns, not the within-subject statistics.

The main analyses described in the paper were based on two GLMs. Unless otherwise specified, we only considered journeys where the participant always moved toward the goal (“optimal” journeys), but other journeys were modeled separately. GLM1 included the following conditions convolved with the canonical HRF basis function: main effect of cue screen; main effect of feedback screen; and main effect of navigation screen for suboptimal journeys. We modeled navigation screens during optimal journeys independently for (1) line changes, (2) exchange stations without a line change, (3) elbow stations, and (4) regular stations without response switch. Additionally, we included the following parametric modulators for regular stations without response switch: distance to goal in number of stations (*D*_*S*_); distance to goal in number of line changes (*D*_*L*_); distance to goal in number of exchange stations (*D*_*X*_); and the U-turn cost (*D*_*U*_). GLM2 included the following conditions: main effect of cue screen; main effect of feedback screen; and main effect of navigation screen. Additionally, the navigation screen included the following parametric modulators: type of station (exchange > regular); type of response (switch > stay); interaction between station and response; distance to goal in number of stations (*D*_*S*_); and performance on the current step (1 if optimal and −1 otherwise).

All effects reported survived FDR correction for multiple comparisons, unless noted in the main text. Images and tables are thresholded at p < 0.001, unless otherwise noted. All the analyses described here focused on effects during the time of navigation. Peak activations are reported with the coordinate system of the MNI template brain. Regions of interest (ROI) were defined by manually selecting clusters under a threshold of p < 0.001 uncorrected.

The mask in rlPFC was extracted from a main effect of distance to goal in GLM2.

### BOLD-RT Correlation Analysis

We extracted the average beta obtained from GLM1, and we obtained average values for dmPFC and PMC. We also obtained similar beta values of effect of D_S_ and D_L_ in explaining log-reaction times (see Experimental Procedures, Behavioral Analyses). We then performed a non-parametric Spearman correlation across participants for each region and type of distance.

### Single-Trial GLM Approach

We performed a single-trial analysis in order to extract the average signal in PMC before and after a line change, an elbow station, an exchange station without response switch (i.e., a line stay), or a regular station without response switch. First, we constructed a design matrix in which each trial was modeled with a unique regressor. From this, we obtained a single scalar BOLD estimate for each voxel on each trial. We then averaged these values within the PMC region for each station type. To avoid double dipping, our ROI was defined based on orthogonal contrast of type of station (exchange > regular) from GLM2 (p < 0.001 uncorrected). Second, we averaged the PMC signal for the neighboring trials around each condition (i.e., line change, elbow station, line stay, and regular station) within the journey. Critically, we restricted these neighboring trials only to regular stations without response switch.

Our prediction was that the BOLD signal in PMC would be higher before a line change than after, but that this difference would not be reflected around elbow stations or exchange stations without a line change. We calculated the difference on the trials immediately before/after each condition and performed a statistical analysis on the main effects of type of station and type of response on this difference. For better visualization, we controlled for between-subject variability in Figure 3A, where we displayed the activity in PMC of all other conditions relative to the average signal in regular stations without response switch.

### Representation Similarity Analysis

For representation similarity analysis (RSA), we constructed a new GLM with four regressors (per scanner run) that each encoded regular stations (without a response switch) corresponding to one subway line (context regressors), and four further parametric regressors that modulated each event by distance to goal (in number of stations; context distance regressors). We used unsmoothed images for this analysis. Additional regressors encoded other quantities (cue screen; feedback screen; in navigation: line changes, “elbow” stations, and exchange stations without a line change; and nuisance regressors). We used a searchlight approach, in which a sphere of 15 mm radius was moved progressively over the brain volume, with the resulting RSA estimates allocated to the centroid voxel for localization and display. Results obtained with a smaller radius (10 mm) were qualitatively very similar. For context decoding (Figure 4B), we estimated for each scanner run (n = 4) the pattern of resulting betas for each of the four context regressors and computed their correlation distance (1-Pearson correlation) yielding a 16 × 16 neural dissimilarity matrix. This matrix was regressed against the predicted representation dissimilarity matrix (RDM) shown in Figure 4A within each searchlight and statistics performed on the resulting betas at the second (between-subject) level. In the predicted RDM, distances were greater between lines than within lines. We excluded comparisons within a single run, to control temporal autocorrelation in the within-session BOLD signal. An identical approach was used for the context distance regressors (Figure 4C). In the control condition (Figure 4D), the assignment of regular and elbow stations to each line was shuffled, so that the hierarchical structure was lost. We then repeated the estimation of beta patterns and the searchlight RSA approach as above.

## Author Contributions

J.B., conception and design, acquisition of data, analysis and interpretation of data, drafting, and revising of the article; D.H. and H.S., conception and design and revising the article; C.S., conception and design, analysis and interpretation of data, drafting, and revising of the article.

## Supplemental Information

Document S1. Figures S1–S4 and Table S3

Table S1. Activations of GLM1, Related to Figure 2 The tables show voxel clusters (larger than five voxels; threshold at 0.001 uncorrected) activated by the multiple contrasts of GLM1 (see Experimental Procedures and Results). The columns indicate (L–R): cluster corrected p value (FDR), cluster size (Voxels), corrected peak p value for each cluster (Peak p), peak t value (Peak t), peak z value (Peak z), and the final column gives the xyz coordinates in MNI space for each peak (Coords). The positive contributions reflect clusters being more active when further away from the goal.

Table S2. Activations of GLM2, Related to Figures 2 and 3 The tables show voxel clusters (larger than five voxels; threshold at 0.001 uncorrected) activated by the multiple contrasts of GLM2 (see Experimental Procedures and Results). The columns indicate (L-R): cluster corrected p value (FDR), cluster size (Voxels), corrected peak p value for each cluster (Peak p), peak t value (Peak t), peak z value (Peak z), and the final column gives the xyz coordinates in MNI space for each peak (Coords).

Document S2. Article plus Supplemental Information
