# Supplementary figures and images for: Neural Mechanisms of Hierarchical Planning in a Virtual Subway Network
Source: Neuron. 2016 May 18;90(4):893–903. doi: 10.1016/j.neuron.2016.03.037 (PMC4882377; doi:10.1016/j.neuron.2016.03.037)

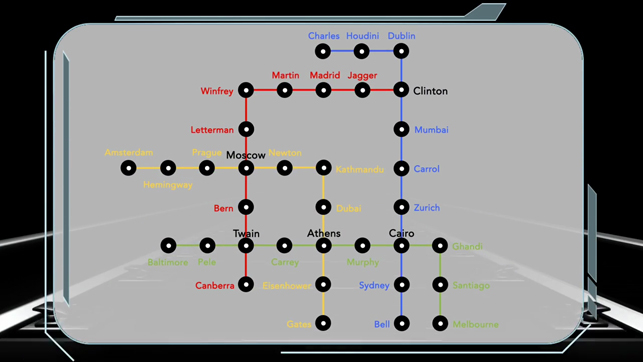

Supplement: Supplementary file 1 [file mmc5.jpg]
