# Supplementary material for: Neural Mechanisms of Hierarchical Planning in a Virtual Subway Network
Source: Neuron. 2016 May 18;90(4):893–903. doi: 10.1016/j.neuron.2016.03.037 (PMC4882377; doi:10.1016/j.neuron.2016.03.037)
Supplement: Document S1. Figures S1–S4 and Table S3 [file mmc1.pdf]

Neuron, Volume *90*

Supplemental Information

Neural Mechanisms of Hierarchical Planning in a Virtual Subway Network

Jan Balaguer, Hugo Spiers, Demis Hassabis, and Christopher Summerfield

Figure S1 (linked to Fig. 2a)

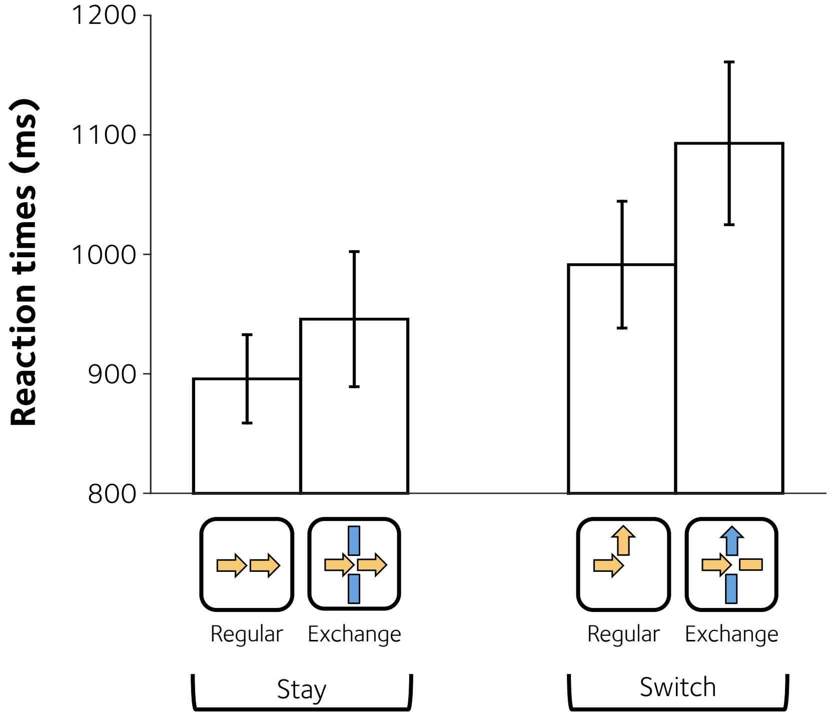

Figure S1. Reaction times (mean ± SEM) for each type of station and response. Participants showed a significant double main effect and were slower when they were required to switch their response; and in exchange stations with more than two possible responses. These two effects added linearly (interaction not significant).

Figure S2 (linked to Fig. 4)

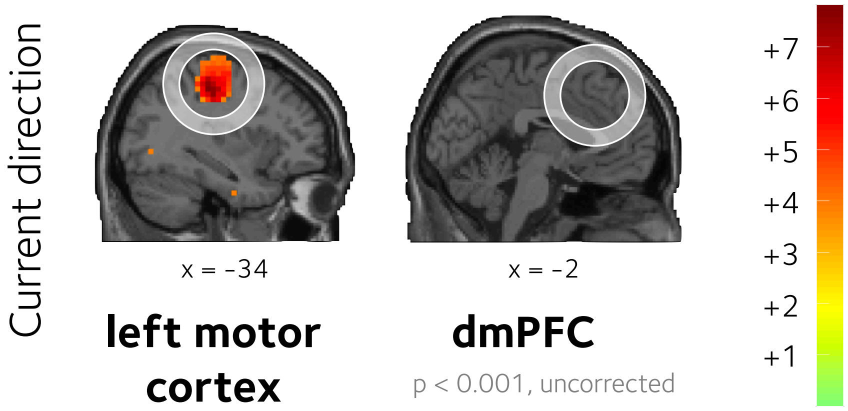

Figure S2. The results of an additional control RSA indentifying voxels encoding the current direction of travel (see **Fig. 4**). In this task, the direction corresponds to the last response given in the current journey (but not including the one given in the current trial). The peak activation was found in left primary motor area. No significant decoding of direction was observed in the dmPFC. This RSA was performed in a very similar manner to the other ones, with the same RDM (see Fig. 4a) but comparing the multivoxel patterns associated with each direction (North, East, West, South) instead of those associated with the current line.

Figure S3 (linked to Figs. 2b, 2c and 3a)

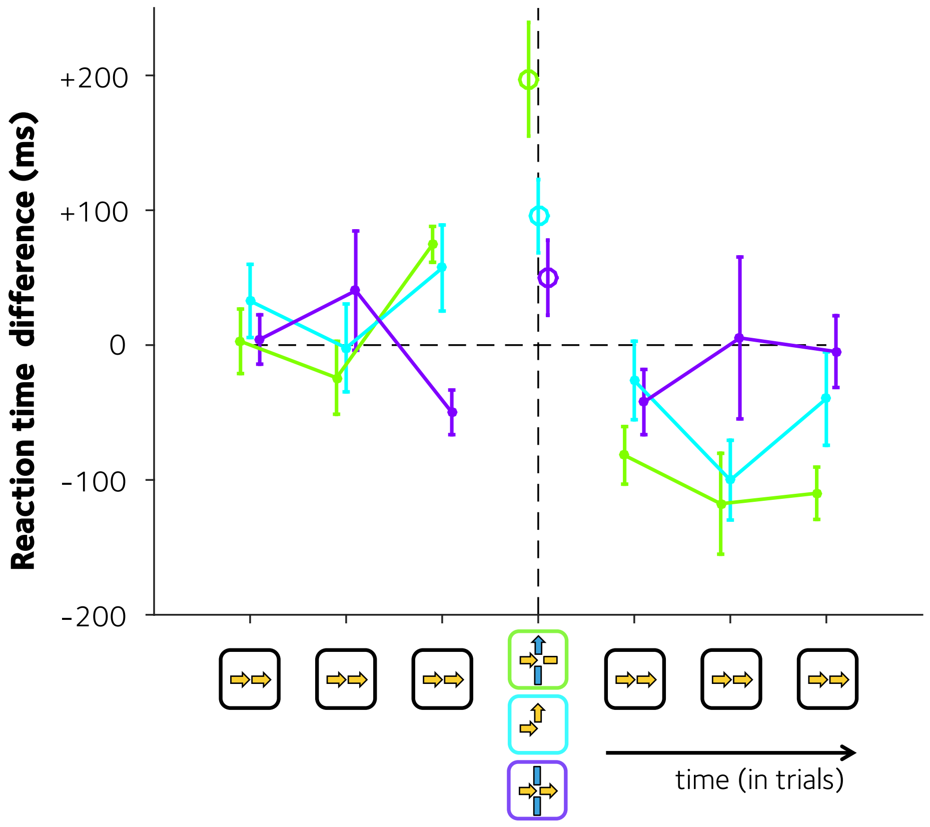

Figure S3. Reaction times (mean ± SEM; relative to average for regular stations) on regular stations (without response switch) over 3 trials that preceded (left points) or followed (right points) a context switch (green), an exchange station without line change (purple) or an elbow station (cyan). Reaction times at the context switch, exchange station or elbow are shown with a single point in the corresponding colour. The average reaction times in regular stations is represented by the horizontal dashed line.

Figure S4 (linked to Fig. 2f)

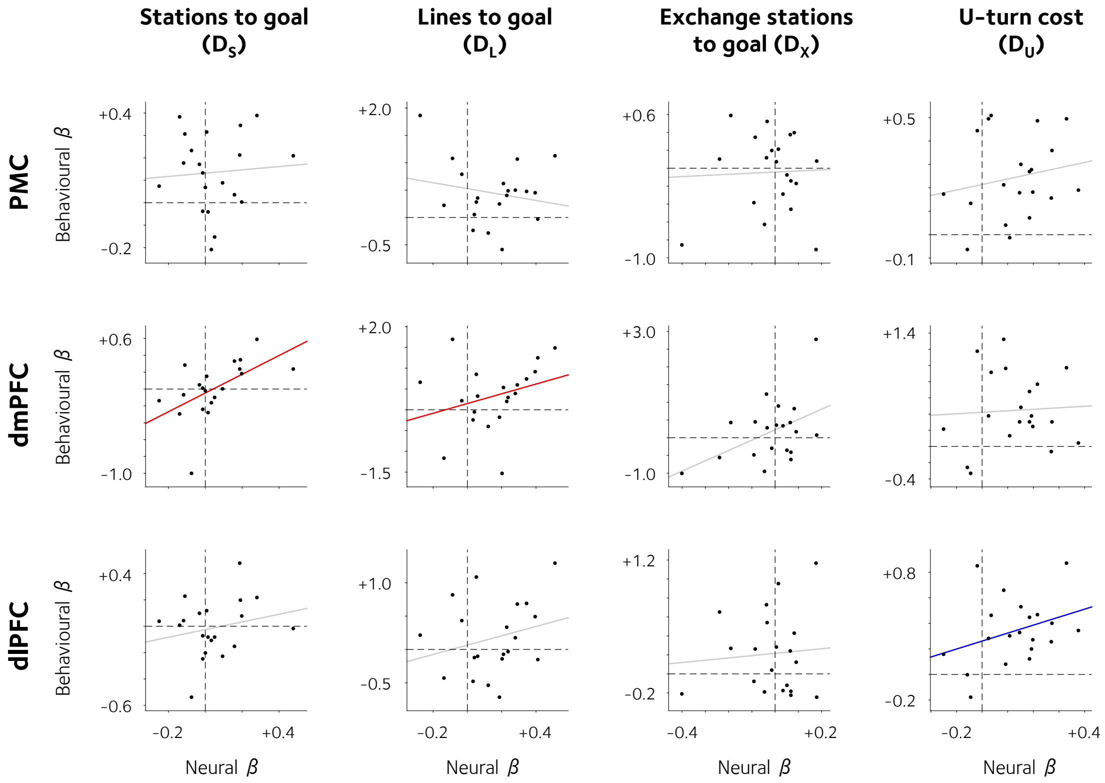

Figure S4. Between-subjects correlation between (i) the regression coefficients that best link distance measures to RT, and (ii) the regression coefficients that described the encoding of distance measures in BOLD signals recorded from the PMC, dmPFC and dlPFC (obtained using GLM1). Best-fitting linear trends are shown for significant (red; $p < 0.05$), marginally significant (blue; $p < 0.05$ one-tailed) and not significant (grey) Spearman correlations.

Table S1 (linked to Fig. 2)
Activations of GLM1

Tables show voxel clusters (larger than 5 voxels; threshold at 0.001 uncorrected) activated by the multiple contrasts of GLM1 (see Methods and Results). Columns indicate (L-R): cluster corrected p-value (FDR), cluster size (Voxels), corrected peak p-value for each cluster (Peak p), peak t-value (Peak t), peak z-value (Peak z) and the final column gives the xyz coordinates in MNI space for each peak (Coords).
Positive contributions reflect clusters being more active when further away from the goal.

[Table S1.xlsx]

Table S2 (linked to Figs. 2 and 3)
Activations of GLM2

Tables show voxel clusters (larger than 5 voxels; threshold at 0.001 uncorrected) activated by the multiple contrasts of GLM2 (see Methods and Results). Columns indicate (L-R): cluster corrected p-value (FDR), cluster size (Voxels), corrected peak p-value for each cluster (Peak p), peak t-value (Peak t), peak z-value (Peak z) and the final column gives the xyz coordinates in MNI space for each peak (Coords).

[Table S2.xlsx]

Table S3 (linked to Figs. 1 and 2)
Correlations of distances

Mean correlation across the cohort between the multiple measures of distance to goal

	D_L	D_X	D_U
D_S	0.6134	0.6958	0.4228
D_L	-	0.5065	0.4087
D_X	-	-	0.1235
